# Evaluation of the Finnish Exposure Notification App “Koronavilkku” According to Key Performance Indicators: Cross-Sectional Study

**DOI:** 10.2196/89472

**Published:** 2026-09-21

**Authors:** Wioleta Kitowska, Mika Pihlajamäki, Sara Wickström, Kaija Tolmunen, Otto Helve, Juha Mykkänen, Timothée Dub, Lotta Siira

**Affiliations:** 1 Field Epidemiology Path (EPIET), ECDC Fellowship Programme, European Centre for Disease Prevention and Control, Stockholm, Sweden; 2 Finnish Institute for Health and Welfare (THL), Mannerheimintie 166, Helsinki, Uusimaa, 00300, Finland, 358 29 524 6000; 3 Gofore Oyj, Helsinki, Finland

**Keywords:** COVID-19, pandemic, cross-sectional, Finland, digital health, mobile app, preparedness

## Abstract

**Background:**

Mobile exposure notification apps (ENAs) can play a significant role in future pandemics. Identifying lessons learned and areas for improvement from such apps, particularly within specific social and cultural contexts, is therefore crucial.

**Objective:**

This study aimed to evaluate the Finnish ENA “Koronavilkku,” according to chosen key performance indicators to assess the ENA’s use and performance during the COVID-19 pandemic in Finland, and to identify strengths and improvement areas.

**Methods:**

Using available data on Finland’s ENA, we defined metrics for four key performance indicators: ENA use extent, enablers and barriers to use, the ENA’s influence on user behavior, and overall acceptability. We performed an evaluation study combining system data and data from a survey of 4061 respondents conducted in April 2022. A sentiment analysis was performed on open-ended responses.

**Results:**

Koronavilkku ENA peak coverage reached approximately 53% of active smartphone users shortly after launch. Use declined over time, particularly among individuals diagnosed with COVID-19. User engagement remained high—among ENA users who received a token, the proportion that entered their token into the ENA was more than 50% during the whole study period. Males, individuals older than 34 years, and those living alone were less likely to use the ENA, while those with higher education and households with children were more likely to be ENA users. Almost 70% (64,642/94,280) of SARS-CoV-2–positive users reported not receiving a token from health authorities. Users reported higher adherence to pandemic guidance than nonusers, with prevalence ratios ranging from 1.24 (95% CI 1.19‐1.29) to 1.65 (95% CI 1.45‐1.91). Descriptive analyses suggested higher reported adherence among partially compliant users after exposure notifications, whereas little change was observed among noncompliant individuals. Overall acceptability was relatively high, with 68% of respondents using it at least once. However, 49% of those users eventually discontinued use. Our sentiment analysis of open-ended feedback suggests that users expressed more positive attitudes toward the ENA compared to nonusers. However, a substantial portion of the feedback reflected neutral or negative sentiments.

**Conclusions:**

Based on observed ENA use trends, we recommend maintaining long-term public interest and engagement in ENAs during prolonged emergencies. Tailored communication should be developed encouraging uptake among demographic groups with lower adoption rates. Initial studies during app rollout could inform these strategies. A major barrier to the ENA’s performance was token issuance; earlier and more systematic automation of token distribution in Finland could have enabled more timely exposure notifications and reduced bottlenecks. Future ENAs should define evaluation criteria during development, with periodic assessments measuring effectiveness and informing improvements. A deeper analysis of open-ended feedback provided for the Koronavilkku ENA using advanced language models could provide additional insights into user perceptions and concerns. By addressing these areas, future digital contact tracing tools may improve uptake, acceptability, and integration into pandemic response efforts.

## Introduction

Finland reported its first imported case of COVID-19 in late January 2020, and by mid-March the number of confirmed cases had risen to over 400. In response, on March 16 the government declared a state of emergency, introducing wide-ranging measures including the closure of schools and public facilities, restrictions on public gatherings, and limitations on cross-border travel. Some of these measures were gradually eased by May 2020, although restrictions were reintroduced in autumn as case numbers increased again. Finland began its vaccination campaign in late December 2020, and by November 2021, approximately 80% of the population aged 12 years and older had been vaccinated. As vaccination coverage expanded and the epidemiological situation improved, restrictions were progressively lifted, with all major measures ending by the end of June 2022 [1-3].

Mobile exposure notification apps (ENAs) using digital proximity tracing were introduced during the COVID-19 pandemic in many countries worldwide. In Finland, the national ENA “Koronavilkku” was launched on August 31, 2020, and discontinued on June 1, 2022. The aim of the Finnish ENA was intended to supplement conventional contact tracing and serve as an early warning system for the individual user in case of potential exposure to a SARS-CoV-2–positive person in order to provide them with behavioral guidance mitigating the chance of further transmission [4]. ENA use was voluntary and free of charge.

The ENA was developed using a decentralized approach. Finnish legislation and the European architecture and protocols used for ENAs placed strong emphasis on privacy, data security, and data safety. Due to these constraints, very limited performance information was gathered through the ENA to describe its potential effectiveness under the stated aims [4]. In addition to the ENA, a dedicated system was provided to all health care provider organizations in Finland to enable token issuance for infected app users, which allowed users to activate exposure notifications to potential contacts. The organizations, in turn, granted system access to health professionals who sent tokens to ENA users.

Several studies have evaluated the uptake, acceptability, and potential impact of ENAs during the COVID-19 pandemic, with mixed findings. Modeling and empirical studies suggest that even moderate uptake may contribute to reducing transmission, although effectiveness depends on multiple factors, including user engagement and timeliness of notifications [5-10]. More recent research has emphasized variability in how effectiveness is defined and measured, as well as the importance of contextual factors in determining impact [11]. Research across European countries has also highlighted variability in adoption and acceptability, with sociodemographic factors, trust, and perceived usefulness influencing uptake [12-15]. Implementation challenges, such as delays in notification processes and integration with public health systems, can limit the overall performance of these tools [16,17]. However, comprehensive evaluations combining system-level performance indicators with user-reported experiences remain limited. Our aim was to evaluate the Finnish ENA according to chosen key performance indicators (KPIs) to gain insight into the ENA’s use and performance during the COVID-19 pandemic in Finland, as well as to identify strengths and areas for improvement.

## Methods

### Overview

We evaluated the ENA during its period of use (August 31, 2020-June 1, 2022) based on the following set of KPIs:

1. extent of ENA use,
2. enablers and barriers to ENA use,
3. influence of the ENA on user behavior, and
4. general acceptability of the ENA.

### Operational Definitions

Active ENA use: the device has the ENA installed, is operational, and communicates at least once every 24 hours with the backend server to retrieve keys (see “key-matching process”).

Keys: randomly generated, encrypted identifiers exchanged through Bluetooth Low Energy between participating devices that are near one another, according to defined distance and duration criteria. These keys provided the basis for anonymous “matching” of devices and the subsequent triggering of exposure notifications.

Key-matching process: phones regularly downloaded keys from users who had received a positive SARS-CoV-2 result and entered their token into the ENA from the backend server and checked them against their own history of contacts for matches. If a match was found, it triggered the exposure notification mechanism based on the calculated exposure risk value (ERV).

Token: one-time code issued by the system used by health care providers upon receiving a positive COVID-19 diagnosis. The token was issued manually to ENA users by health care professionals, although automatic issuance was introduced in some parts of Finland from June 2021. When entered into the ENA by the user, the user’s keys were sent to the backend server. Entering a token locked the ENA and it no longer tracked exposures.

Exposure notification: a message issued by the ENA alerting the user that they had recently been in proximity to an individual who has received a positive SARS-CoV-2 finding and has shared their result via the app.

### Data Collection

We accessed system activity data from the ENA provider and the ENA’s backend. In Finland, the ENA providers were the Social Insurance Institution (Kela) and the Finnish Institute for Health and Welfare (THL). Variables retrieved included: number of downloads (data available for August 31, 2020-February 8, 2021), number of active users (data available for December 7, 2020-April 24, 2022), number of tokens issued (data available for September 7, 2020-May 29, 2022), and number of tokens used (data available for September 7, 2020-May 29, 2022).

Furthermore, we used data from the cross-sectional online survey conducted from April 13 to April 22, 2022, by a Finnish polling company among respondents aged 15‐79 years, based on a convenience sample of the company’s and its subcontractors’ panelists. The survey included 4061 respondents, corresponding to approximately 0.07% of the Finnish population (~5.5 million). Weights were assigned to all respondents based on the distribution of the Finnish population with regard to sex, age, and geographic area (data from Statistics Finland – population by sex and age and major regions in mainland Finland as of December 31, 2020).

Variables from the survey included respondents’ sociodemographic characteristics, ENA use, ENA removal, reasons for nonactivation, reasons for removal, receipt of exposure notifications, token receipt, speed of token delivery, use of tokens, adherence to recommendations after notification, reasons for discontinuation of adherence to recommendations, consulting a health care provider after exposure notification, general behavior during the pandemic, COVID-19 diagnosis, and overall feedback on the ENA.

We used data on population size by sex, age, region, and year during 2020‐2022 from Statistics Finland. We retrieved the daily number of COVID-19 cases reported to the National Infectious Diseases Register (NIDR) from August 31, 2020, to June 1, 2022 [18].

### Koronavilkku Survey Variables

**Figure 1.**
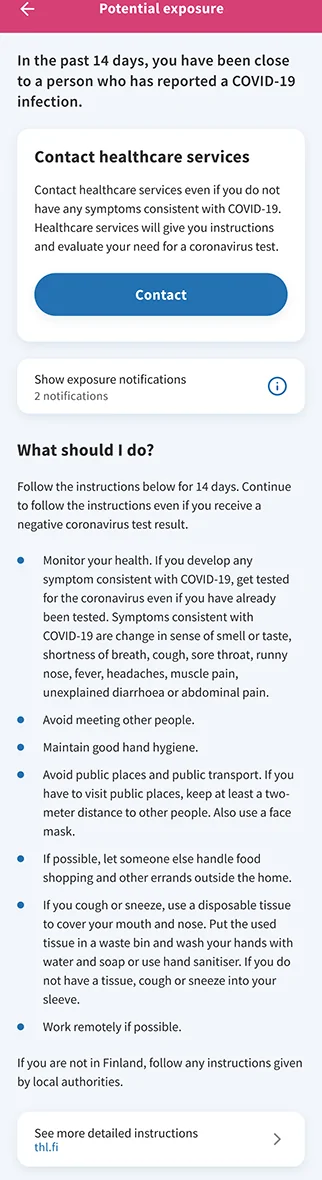
Guidance shown to users of the Finnish exposure notification app upon receipt of an exposure notification from March 4, 2021, to June 16, 2021. The instructions were updated in line with the national guidelines [4].

We created the following terms based on variables from the Koronavilkku survey:

- ENA user or nonuser: we divided respondents into two main groups: ENA users and nonusers based on the question “Have you downloaded the Koronavilkku application on your phone?” ENA users were further classified as reporting current or previous use of the ENA. Nonusers were classified as reporting never using the ENA or downloading the ENA but never activating it.
- Exposure notification guidance*:* along with the exposure notification, the ENA gave guidance that was in line with general national pandemic guidelines (Figure 1). Based on the guidance, we used the following terms: self-observation of well-being, avoiding social interactions, hand hygiene, wearing a mask, avoiding errands, cough hygiene, working from home.

The survey included two questions about adherence to the above-mentioned exposure notification guidance: general behavior during the pandemic and after receiving an exposure notification. Respondents were able to mark their adherence to each of the seven points on a scale of “never, part-time, entire time, don’t recall/unsure.”

### Data Analysis

We assessed each of the four KPIs (extent of ENA use, enablers and barriers to ENA use, ENA influencing user behavior, and general acceptability) according to a set of predefined metrics specified in Multimedia Appendix 1. The analysis was performed using R software (version 4.2.1; R Foundation for Statistical Computing) in RStudio (Posit Software, PBC). *P* values less than .05 were considered significant.

This study used two main data sources for analysis: (1) ENA backend data, used to describe the extent of ENA use; and (2) data from the Koronavilkku survey, used to assess enablers and barriers to ENA use, ENA influence on user behavior, and general acceptability. The data sources for each metric are specified in Multimedia Appendix 1.

Survey weights based on age, sex, and region were applied to descriptive analyses of survey data (excluding sentiment analysis) to improve representativeness of the Finnish population. Prevalence ratios (PRs) were estimated using log-binomial regression models fitted to unweighted data. A large language model (LLM) was used for sentiment analysis of open-ended feedback in the survey. The sentiment analysis was performed using Python 3 (version 3.10.12, Python Software Foundation) and the Llama model version 3.1 70B (model identifier: llama3.1:70b-instruct-q4_K_M; Meta). Finnish input texts were analyzed natively without translation. The response was classified using a prompt that asked for feedback to be in one of four categories: positive, neutral, negative, and not understandable feedback. Prompt rules asked it to return only one of these categories and never to output the original clause or anything other than the classes.

### Sentiment Analysis Validation

To compare Llama classifications to human classifications, we randomly sampled 100 feedback responses, and one reviewer added manual annotations about human-evaluated sentiment. Overall agreement was 63% (Cohen κ=0.486) and considered “moderate.” The main pattern found was that Llama classified positives and negatives well but misclassified many human-labeled neutrals as “not understandable feedback.” These were mostly policy or feature suggestions without clear opinion on the current version or just statements that the users did not have anything to say.

The validation results can be found in Table S1 in Multimedia Appendix 2.

### Generative AI

Generative AI was used solely as an analytic tool for sentiment classification (LLM) and was not used to draft or edit the paper.

### Ethical Considerations

The app user data were collected and analyzed as part of THL’s mandate; therefore, no ethical approval was required for this study. Upon being recruited to the survey study, participants gave their informed consent. The participants did not receive remuneration for their participation; the polling company had a monthly lottery, where participants were eligible to win prizes, such as gift cards. All analysis was performed on anonymized data; no personal identifying data was used. The legal basis for the ENA and THL’s role related to it is detailed in the Communicable Diseases Act 1227/2016 HE 101/2020.

## Results

### Extent of ENA Use

The extent of ENA use was assessed based on backend ENA data.

**Figure 2.**
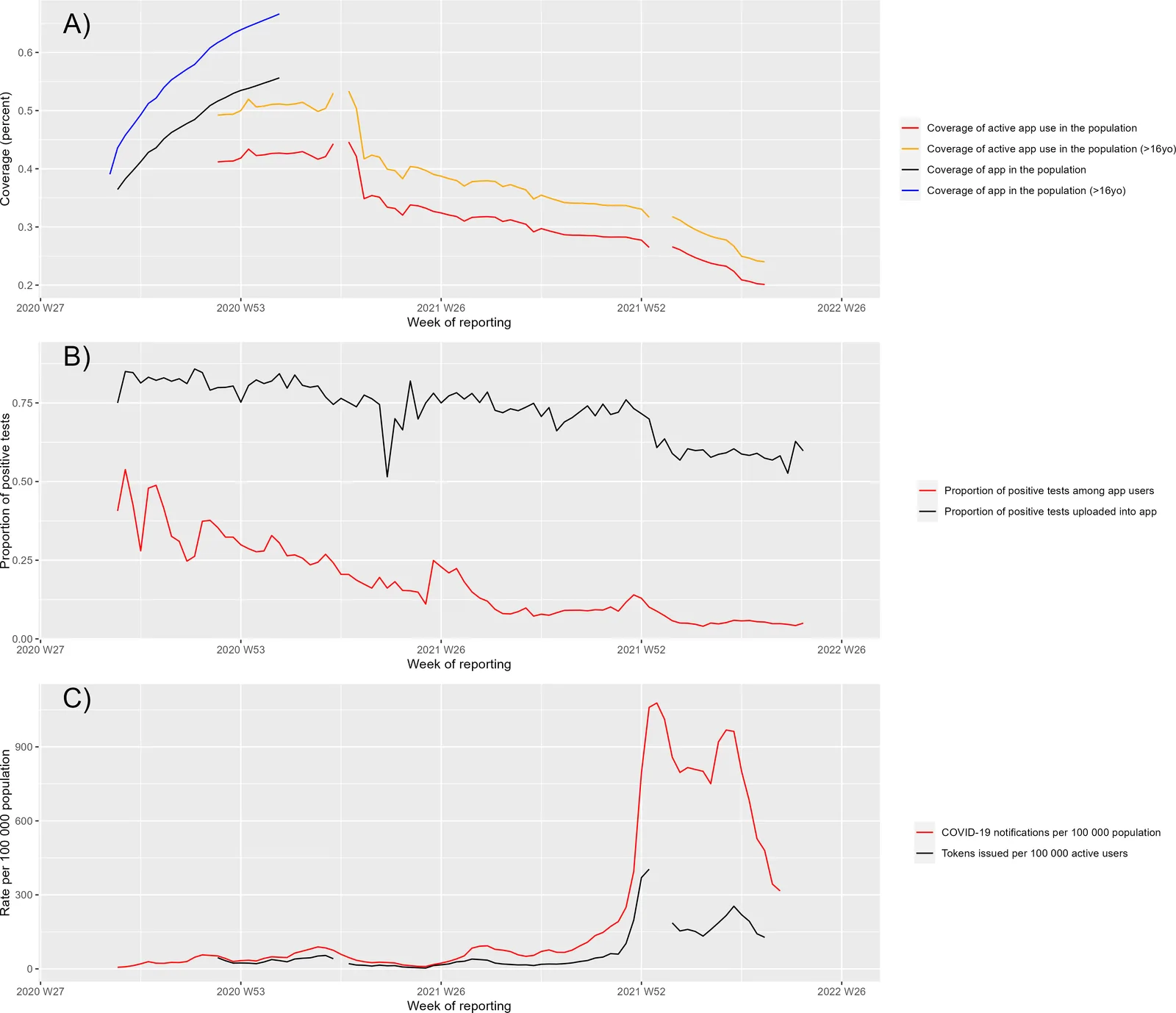
(A) Proportion of the population that have downloaded and actively use the exposure notification app in Finland August 31, 2020, to April 24, 2022. Results shown for the whole population of Finland and for the population of Finland >16 years old. (B) Proportion of positive SARS-CoV-2 tests among ENA users among all SARS-CoV-2 positive tests in Finland August 31, 2020, to April 24, 2022; proportion of positive SARS-CoV-2 tests uploaded into the ENA (used tokens) among all ENA users that received a token (issued tokens) in Finland August 31, 2020, to April 24, 2022. (C) Rate of SARS-CoV-2 notifications per 100,000 population and rate of tokens issued per 100,000 active users.

We used the number of downloads as a proxy for active ENA use at the introduction of the app, after which we switched to monitoring active ENA use. In late 2020, the ENA was actively used by around 40%‐50% of the Finnish population. In 2021, the active coverage peaked around weeks 12‐14 at 53% among the Finnish population older than 16 years old (45% in the general population), after which it declined to less than 30% in early 2022 (Figure 2A).

At all times during the pandemic, fewer than 50% of all people who tested positive for COVID-19 both used the ENA and entered their tokens. The proportion was highest at the launch of the ENA but declined to less than 30% before the start of 2021. In total, 94,280 tokens were issued during the ENA’s lifetime, of which 69% (64,642/94,280) were entered into the ENA (Figure 2B).

The weekly proportion of ENA users who received a token and entered it into the ENA to alert other users of potential exposure reached 86% (586/683) in late 2020 and did not dip below 50% at any point. During the period of ENA use, the mean was 73% (SD 9%) and the median 75% (IQR 67%-80%), indicating high user engagement (Figure 2B).

The rate of positive tests among ENA users relative to the rate of positive tests reported in the general population was similar at the beginning, when the COVID-19 notification rate was relatively low. The rate of tokens issued did not scale in the same way as the notification rate during its highest peaks (Figure 2C).

### Enablers and Barriers to ENA Use

Enablers and barriers to ENA use were assessed based on data from the Koronavilkku survey.

#### Sociodemographic Characteristics of ENA Users vs. Non-ENA Users

The prevalence of ENA nonusers was higher among males than females (PR 0.93, 95% CI 0.89‐0.97) and in the age groups above 34 years. There were no significant regional differences between ENA users and nonusers (Table 1).

**Table 1.** Sociodemographic characteristics of exposure notification app users vs non-exposure notification app users based on a survey conducted in Finland from April 13 to 22, 2022 (n=4061, weighted n=4334). Results weighted according to age, sex, and regional distribution of the Finnish population.

Characteristics	Non-ENA^a^ users, (N=1321), n (%)	Non-ENA users, Weighted (n=1407)^b^	ENA users, (N=2740), n (%)	ENA users, Weighted (n=2927)^b^	PR^c^	95% CI	*P* value
Sex							
Female	617 (47)	46% (43.3‐48.8)	1441 (53)	51.9% (50‐53.8)	Ref.	—^d^	—
Male	704 (53)	54% (51.2‐56.7)	1299 (47)	48.1% (46.2‐50)	0.93	0.89‐0.97	<.001
Age group (years)							
15‐24	140 (11)	11.3% (9.7‐13.2)	397 (14)	15.2% (13.8‐16.6)	Ref.	—	—
25‐34	189 (14)	13.8% (12.1‐15.8)	495 (18)	17.4% (16.1‐18.9)	0.98	0.91‐1.05	.54
35‐49	394 (30)	25.3% (23.1‐27.6)	741 (27)	22.8% (21.4‐24.4)	0.88	0.83‐0.94	<.001
50‐64	354 (27)	27.2% (24.9‐29.7)	639 (23)	23.3% (21.8‐25)	0.87	0.81‐0.93	<.001
65‐79	244 (18)	22.3% (19.9‐24.9)	468 (17)	21.2% (19.6‐23)	0.89	0.83‐0.96	.002
Region							
Southern Finland	292 (22)	20.9% (18.8‐23.2)	594 (22)	21% (19.5‐22.6)	Ref.	—	—
Helsinki-Uusimaa	385 (29)	28.4% (26‐30.9)	910 (33)	32.6% (30.9‐34.4)	1.05	0.99‐1.11	.11
Western Finland	332 (25)	25.8% (23.5‐28.3)	659 (24)	24.4% (22.8‐26)	0.99	0.93‐1.06	.8
Northern and Eastern Finland	312 (24)	24.9% (22.5‐27.4)	577 (21)	22% (20.4‐23.6)	0.97	0.91‐1.04	.34
Education							
Primary or national school	212 (16)	16.5% (14.6‐18.7)	283 (10)	10.8% (9.6‐12)	Ref.	—	—
High school	180 (14)	13.5% (11.7‐15.5)	417 (15)	15.1% (13.8‐16.5)	1.22	1.12‐1.34	<.001
Vocational, technical or business school	435 (33)	32.5% (30‐35.1)	679 (25)	24.6% (23‐26.3)	1.07	0.98‐1.17	.16
University of applied sciences	173 (13)	12.7% (11‐14.6)	431 (16)	15.1% (13.8‐16.5)	1.25	1.14‐1.37	<.001
College	137 (10)	10.8% (9.2‐12.7)	348 (13)	13.2% (12‐14.6)	1.26	1.14‐1.38	<.001
University or college	184 (14)	14% (12.2‐16)	582 (21)	21.2% (19.7‐22.8)	1.33	1.22‐1.45	<.001
Occupation							
Employee	386 (30)	28.5% (26.1‐31.1)	727 (27)	25.3% (23.7‐26.9)	Ref.	—	—
Functionary	70 (5.4)	5.2% (4.1‐6.5)	288 (11)	10% (9‐11.2)	1.23	1.15‐1.31	<.001
Higher functionary, expert	90 (7)	6.7% (5.5‐8.2)	339 (13)	11.9% (10.7‐13.1)	1.21	1.13‐1.29	<.001
Leading position, entrepreneur	80 (6.2)	6.1% (4.9‐7.6)	250 (9.2)	9% (7.9‐10.1)	1.16	1.07‐1.25	<.001
Pensioner	326 (25)	28.2% (25.7‐30.9)	584 (22)	25% (23.3‐26.8)	0.98	0.92‐1.05	.59
Stay-at-home-parent	31 (2.4)	2.4% (1.7‐3.4)	33 (1.2)	1.2% (0.8‐1.7)	0.79	0.6‐0.98	.06
Student	108 (8.4)	8.3% (6.9‐10)	286 (11)	10.5% (9.4‐11.8)	1.11	1.03‐1.19	.005
Unemployed	193 (15)	14.2% (12.4‐16.1)	187 (6.9)	6.6% (5.8‐7.6)	0.75	0.67‐0.84	<.001
Other	5 (0.4)	0.4% (0.1‐0.9)	15 (0.6)	0.5% (0.3‐0.9)	1.15	0.82‐1.39	.29
Unknown	32	—	31	—	—	—	—
Household situation							
Single	532 (40)	40.2% (37.6‐43)	917 (33)	33.9% (32.1‐35.7)	Ref.	—	—
Couple	298 (23)	23.2% (21‐25.6)	684 (25)	25.6% (24‐27.4)	1.10	1.04‐1.16	.001
Household with children	257 (19)	18% (16‐20.1)	685 (25)	23% (21.4‐24.5)	1.15	1.09‐1.21	<.001
Other adult household	234 (18)	18.6% (16.5‐20.8)	454 (17)	17.5% (16.1‐19)	1.04	0.97‐1.11	.22

a ENA: exposure notification app.

b weighted % (95% CI).

c PR: prevalence ratio.

d N/A: not applicable.

ENA users had a higher level of education more often than nonusers. Compared to those that had primary or national school-level education, ENA use was more prevalent among those that finished high school (PR 1.22, 95% CI 1.12‐1.34), a university of applied sciences (PR 1.25, 95% CI 1.14‐1.37), college (PR 1.26, 95% CI 1.14‐1.38), or university or college (PR 1.33, 95% CI 1.22‐1.45; Table 1).

Compared to employees, ENA use was more prevalent among those holding functionary, higher functionary, and leadership positions as well as among students. Nonusers were more often unemployed (PR 0.75, 95% CI 0.67‐0.84; Table 1).

ENA users were more often couples (PR 1.10, 95% CI 1.04‐1.16) or living in households with children (PR 1.15, 95% CI 1.09‐1.21; Table 1).

#### Reasons for Nonactivation of the ENA

**Figure 3.**
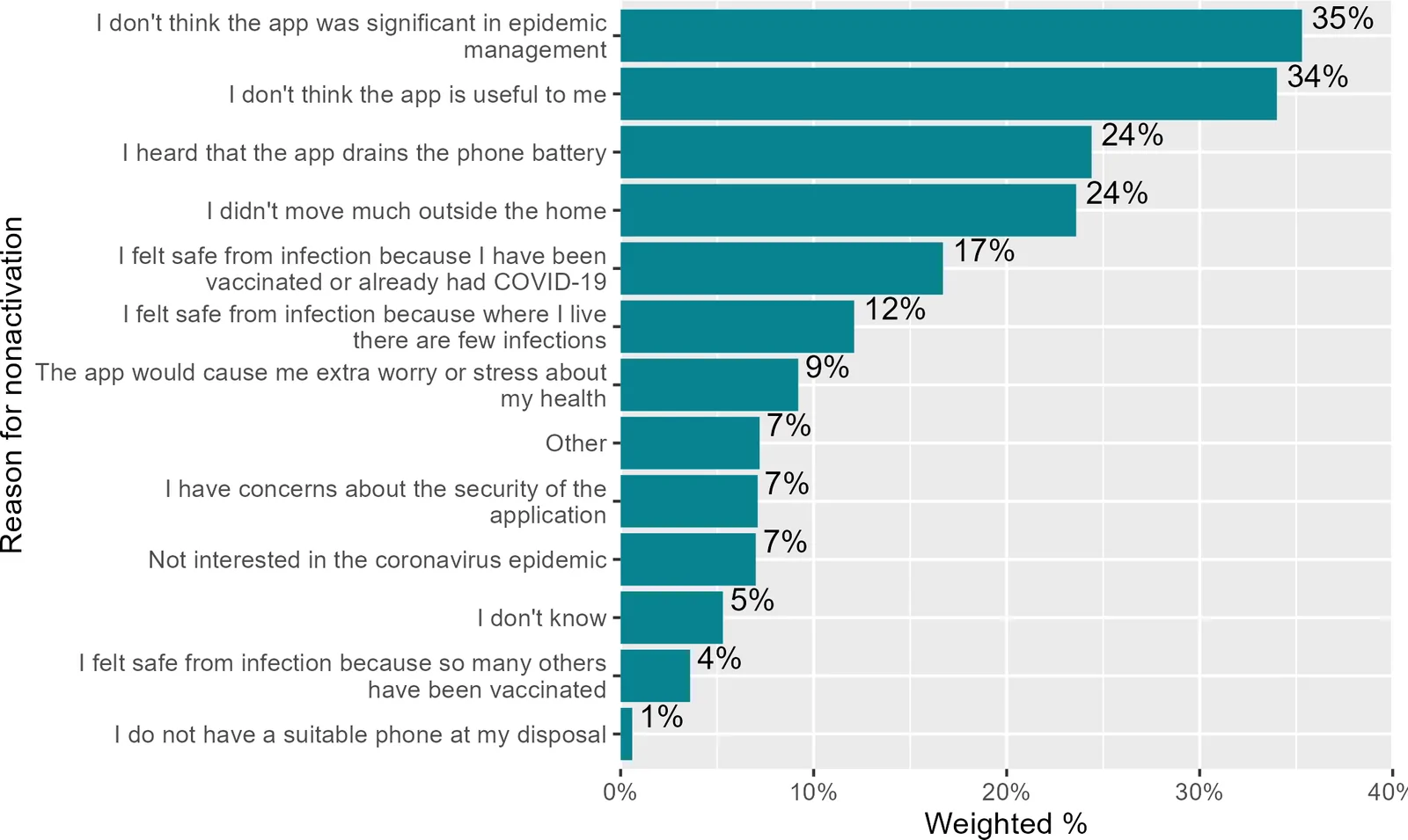
Weighted frequency distribution of reasons for not activating the exposure notification app (n=149, weighted n=163). Data from survey conducted in Finland from April 13‐22, 2022. Results weighted according to age, sex, and regional distribution of the Finnish population. Exact estimates with 95% CIs are provided in Table S2 in Multimedia Appendix 2.

The most frequent reasons for not activating the ENA after download were perceived lack of usefulness of the ENA for pandemic control (35%) or for oneself (34%). Around 24% of respondents indicated phone battery life and staying at home as reasons for nonactivation (Figure 3).

#### General Behavior During Pandemic of ENA Users vs Non-ENA Users

ENA users reported at least part-time adherence to general pandemic guidance more often than nonusers, ranging from avoiding errands (PR 1.24, 95% CI 1.19‐1.29) to wearing a mask (PR 1.65, 95% CI 1.45‐1.91; Table 2). However, these associations may reflect self-selection and cannot be interpreted as causal.

**Table 2.** Adherence to exposure notification guidance of exposure notification app users compared to non-exposure notification app users (n=4061, weighted n=4334). Results weighted according to age, sex, and regional distribution of the Finnish population.

Exposure notification guidance	Non-ENA^a^ users^b^	Non-ENA users adhering (yes)^c^, n (%)	Non-ENA users adhering weighted (yes)^d^	ENA users^b^	ENA users adhering (yes)^c^, n (%)	ENA users adhering weighted (yes)^d^	PR^e^	95% CI	*P* value (chi-square test)
Working from home	1254/1333	452 (36)	35.5% (32.9‐38.2)	2646/2821	1488 (56)	55.1% (53.1‐57)	1.30	1.24‐1.36	<.001
Self-observation of well-being	1281/1365	937 (73)	72.8% (70.3‐75.3)	2687/2869	2307 (86)	85.1% (83.7‐86.5)	1.35	1.26‐1.46	<.001
Avoiding social interactions	1297/1381	986 (76)	76.3% (73.9‐78.6)	2705/2889	2340 (87)	86.3% (84.9‐87.6)	1.30	1.21‐1.41	<.001
Wearing a mask	1310/1395	1142 (87)	87.6% (85.7‐89.3)	2716/2900	2594 (96)	95.6% (94.7‐96.3)	1.65	1.45‐1.91	<.001
Hand hygiene	1298/1383	1182 (91)	91.5% (89.8‐92.9)	2709/2893	2601 (96)	96.1% (95.3‐96.8)	1.43	1.25‐1.65	<.001
Avoiding errands	1297/1383	232 (18)	18% (16‐20.3)	2693/2878	849 (32)	31.2% (29.5‐33)	1.24	1.19‐1.29	<.001
Cough hygiene	1288/1372	1183 (92)	92.1% (90.5‐93.4)	2700/2884	2584 (96)	95.7% (94.9‐96.4)	1.31	1.16‐1.5	<.001

a ENA: exposure notification app.

b n/weighted N.

c Reported at least part time adherence.

d weighted % (95% CI).

e PR: prevalence ratio.

#### Token Use

Based on survey data, of those ENA users reporting a positive SARS-CoV-2 result (n=881, weighted n=904), close to 70% did not receive a token and 1% reported being diagnosed abroad. Of the 30% receiving a token (n=259, weighted n=269), most (79%) reported entering it into the ENA. For approximately 72% of users, tokens were delivered in under 48 hours from diagnosis, and of those, 51% were received within 24 hours. However, delay in token delivery by 3‐4 days was reported by 17%, 5‐7 days by 3%, and over 7 days by 2%. Six percent did not recall the speed of token delivery.

These survey-based token-use estimates are not directly comparable with token use proportions calculated based on ENA backend data, as the backend metrics reflect all eligible users over the full study period, whereas survey metrics reflect self-reported experiences in a cross-sectional respondent sample.

#### Influence of ENA on User Behavior

Influence of ENA on user behavior was assessed based on data from the Koronavilkku survey.

#### Adherence to Recommendations After Notification

**Figure 4.**
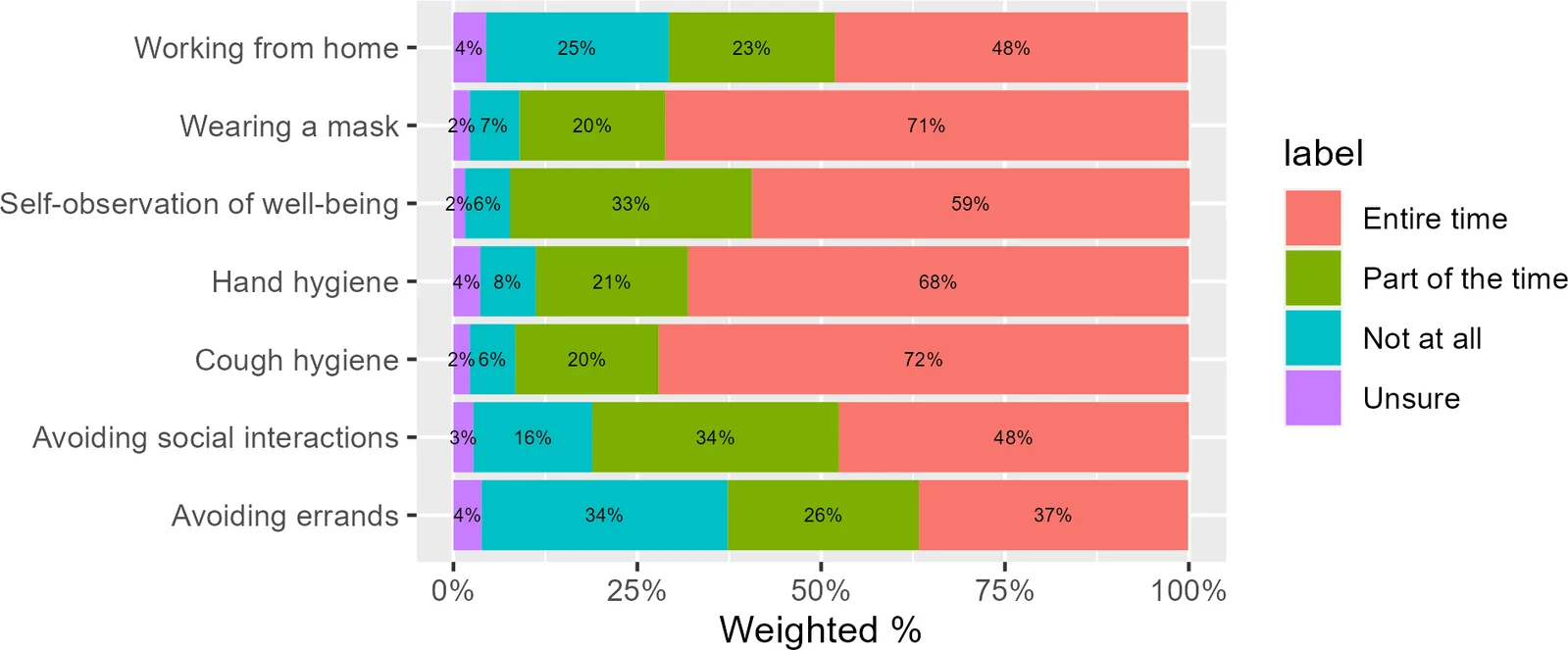
Weighted frequency distribution of adherence to pandemic behavioral recommendations after receiving an exposure notification in the exposure notification app (n=644, weighted n=670). Data from survey conducted in Finland from April 13 to 22, 2022. Results weighted according to age, sex, and regional distribution of the Finnish population. Exact estimates with 95% CIs are provided in Table S3 in Multimedia Appendix 2.

The majority of ENA users who received a notification of potential exposure adhered at least part-time to all guidance provided in the ENA. The least adhered to recommendations were “to avoid running errands” (34% reported no adherence), “work from home” (25% no adherence), and “avoid social interactions” (16% no adherence; Figure 4).

#### Change in Adherence to Recommendations in General During Pandemic vs After Notification

Looking at ENA users (n=2740, weighted n=2927), Bowker’s test of symmetry showed significant differences between general adherence to pandemic recommendations versus after receiving an exposure notification for working from home (*χ*²_3_=75.5; *P*<.001), self-observation of well-being (*χ*²_3_=18.3; *P*<.001), avoiding social interactions (*χ*²_3_=114.6; *P*<.001), wearing a mask (*χ*²_3_=26.9; *P*<.001), and avoiding errands (*χ*²_3_=74.4; *P*<.001).

Adherence to hand hygiene (*χ*²_3_=0.6; *P*=.90) and cough hygiene (*χ*²_3_=0.6; *P*=.90) guidelines showed no differences.

Further analysis of transition patterns in adherence to pandemic recommendations suggested the following:

1. More transitions from none to full-time adherence for “avoiding errands” (odds ratio [OR] 6.08).
2. Transitions from part-time to full-time adherence were more frequent than the reverse for “working from home” (OR 3.53), “self-observation” (OR 2.07), “avoiding social interactions” (OR 6.05), “wearing a mask” (OR 2.5), “avoiding errands” (OR 4.04).
3. Transitions from part-time to no adherence were observed for “working from home” (OR 5.61), “avoiding social interactions” (OR 2.19).

These transition ORs are descriptive and should not be interpreted as inferential tests of specific pairwise changes.

#### Reasons for Discontinuation of Adherence to Recommendations

**Figure 5.**
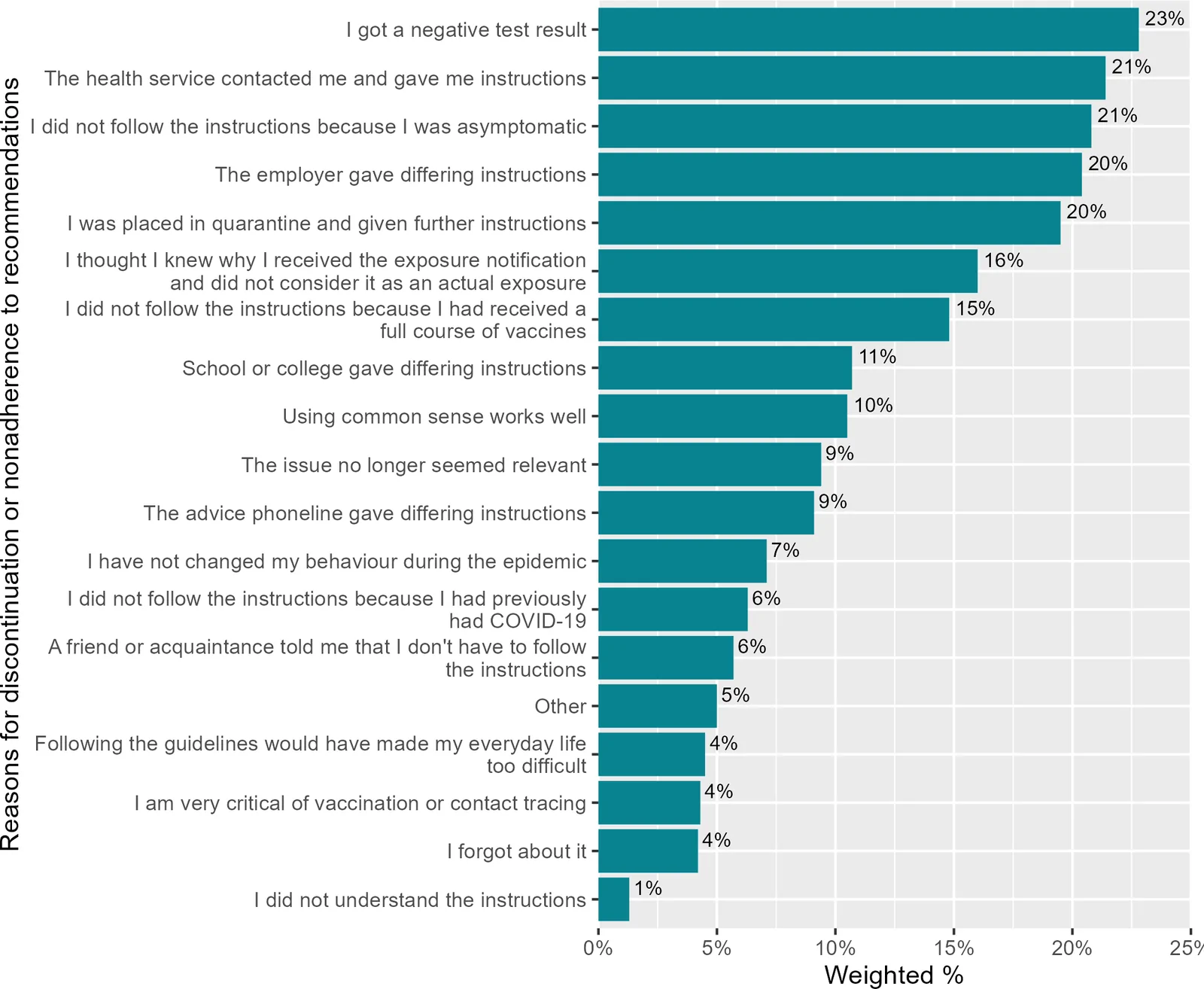
Weighted frequency distribution of reasons for discontinuation or nonadherence to pandemic behavioral recommendations after receiving an exposure notification in the exposure notification app (n=534, weighted n=556). Data from survey conducted in Finland from April 13 to 22, 2022. Results weighted according to age, sex, and regional distribution of the Finnish population. Exact estimates with 95% CIs are provided in Table S4 in Multimedia Appendix 2.

A negative test was the most stated reason for not adhering, partly adhering, or discontinuing adherence to exposure notification guidance (23%). Other commonly reported reasons included receiving instructions from health care (21%), being asymptomatic (21%), receiving instructions from employer (20%), or being put in mandatory quarantine as defined by the Communicable Diseases Act (20%; Figure 5).

#### Contacting Health Care Provider

Among ENA users receiving an exposure notification (n=644, weighted n=670), more than 50% contacted health care afterwards either by phone (25%) or through the Finnish electronic service platform providing symptom checks and guidance called “Omaolo” (DigiFinland Oy; 27%). Around 45% did not report contacting health care and the remaining 3% were unsure.

#### Acceptability of the ENA

The ENA’s acceptability was assessed based on data from the Koronavilkku survey.

#### ENA Use

Among 4061 respondents (weighted n=4334), 33% never downloaded or activated the ENA. Another 34% downloaded and actively used it at the time of the survey, while 33% downloaded the ENA but later removed it or did not activate it again on a new mobile phone.

#### Reasons for Removal

**Figure 6.**
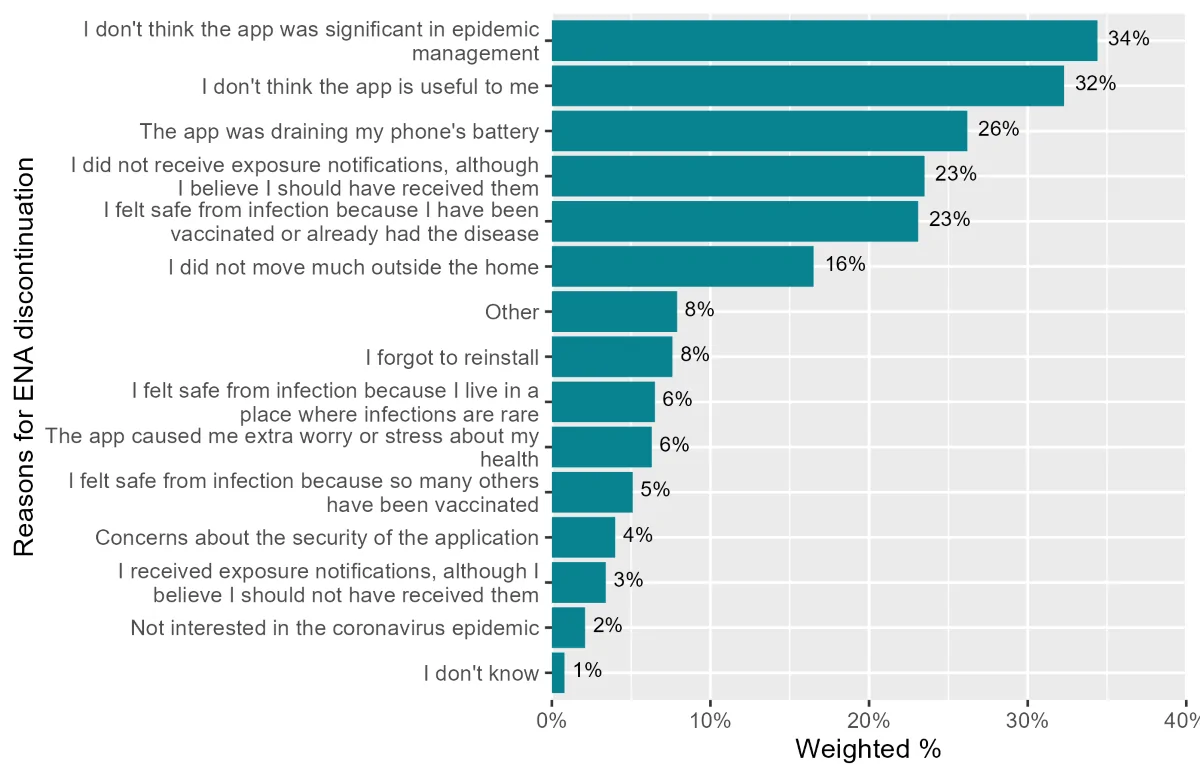
Weighted frequency distribution of reasons for discontinuation of ENA use (n=1370, weighted n=1444). Data from Koronavilkku survey conducted in Finland from April 13‐22, 2022. Results weighted according to age, sex, and regional distribution of the Finnish population. Exact estimates with 95% CIs are provided in Table S5 in Multimedia Appendix 2. ENA: exposure notification app.

Among former ENA users, the most common reasons for discontinuation of ENA use included not seeing any use for pandemic control (34%) or to oneself (32%). Negative effect on battery life was the third most common reason (26%; Figure 6).

#### Overall Feedback

**Figure 7.**
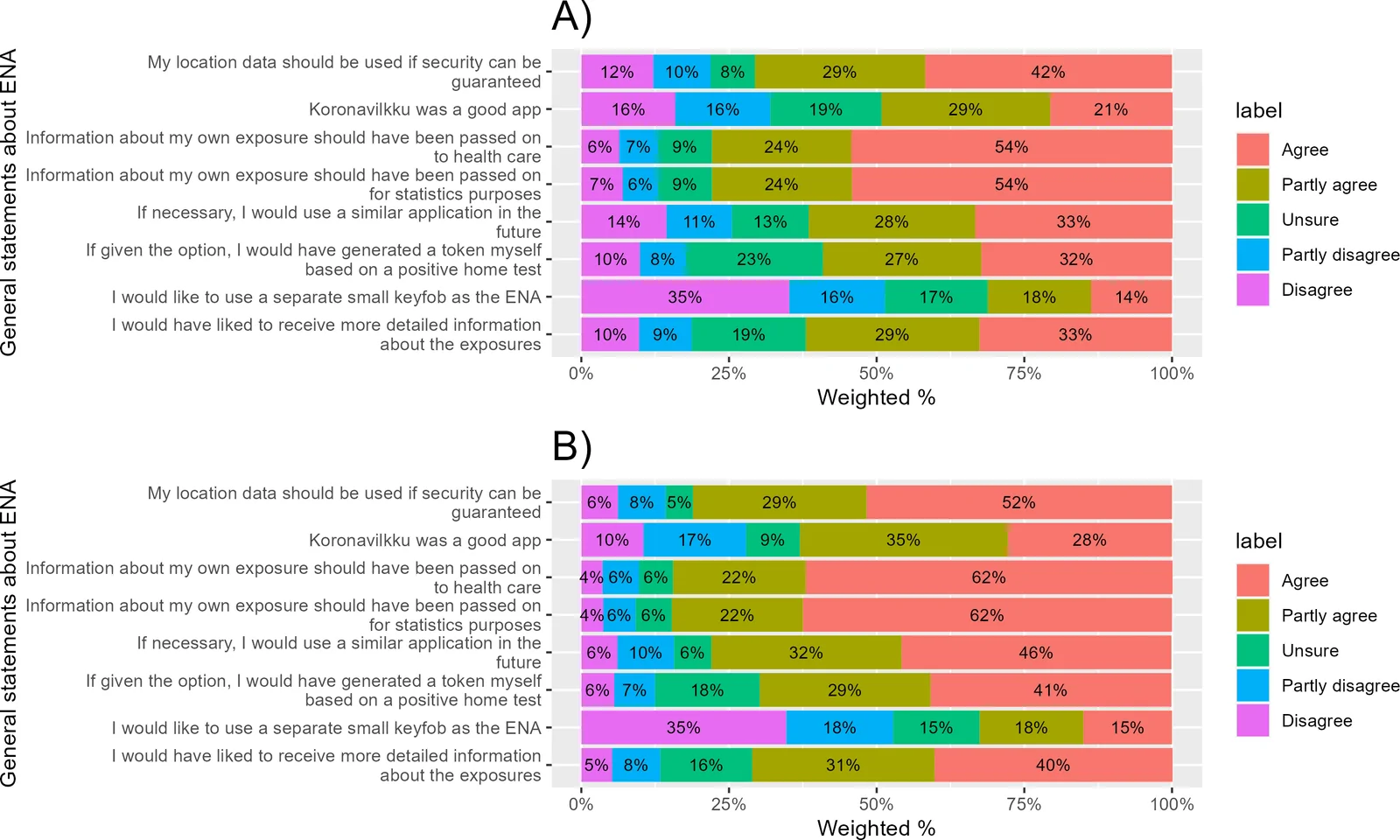
Weighted frequency distribution of attitude toward general statements about the ENA among (A) all respondents (n=4061, weighted n=4334); (B) among ENA users (n=2740, weighted n=2927). Data from Koronavilkku survey conducted in Finland from April 13‐22, 2022. Results weighted according to age, sex, and regional distribution of the Finnish population. Exact estimates with 95% CIs are provided in Table S6 in Multimedia Appendix 2. ENA: exposure notification app.

Opinions on the quality of the ENA were divided. Almost half (49%) of the respondents at least partly agreed with the statement “Koronavilkku was a good application.” Almost two-thirds of respondents (62%) at least partly agreed that they would use the ENA if it was relaunched (Figure 7A).

Among ENA users, this percentage was higher: 63% at least partly agreed that it was a good ENA, and 78% stated that they would use it again (Figure 7B).

Between 62%‐78% of respondents stated they would have accepted more data (such as additional information about exposure or localization data) being collected by the ENA or being sent to notify health authorities and for situation monitoring or statistical analyses (Figure 7A). The percentage of respondents who at least partly agreed with all statements was higher among ENA users (Figure 7B).

#### Sentiment Analysis

Sentiment analysis using an LLM was performed for open-ended feedback provided by 813 respondents, 557 (69%) of whom were ENA users.

In comparison to the survey respondents, open-ended feedback was provided more often by males, aged 15‐24 years, and persons living in Helsinki-Uusimaa and Western Finland regions (Table 3).

**Table 3.** Characteristics of respondents giving open feedback vs those responding to survey overall.

Characteristics	Open feedback respondents (n=813), n (%)	Survey respondents (N=4061), n (%)	*P* value^a^
ENA^b^ use	557 (69)	2740 (67)	.51
Sex			<.001
Female	341 (42)	2058 (51)	
Male	472 (58)	2003 (49)	
Age group (years)			<.001
15‐24	148 (18)	537 (13)	
25‐34	139 (17)	684 (17)	
35‐49	224 (28)	1135 (28)	
50‐64	183 (23)	993 (24)	
65‐79	119 (15)	712 (18)	
Region			<.001
Southern Finland	162 (20)	886 (22)	
Helsinki-Uusimaa	300 (37)	1295 (32)	
Western Finland	207 (25)	991 (24)	
Northern and Eastern Finland	144 (18)	889 (22)	

a Pearson chi-squared test with simulated *P* value (based on 2000 replicates).

b ENA: exposure notification app.

Sentiment analysis determining the emotional tone of the open-ended feedback shows that most provided feedback had a negative sentiment (316/813, 39%), while positive-oriented feedback was provided by 19% (151/813) of respondents. Overall, 12% (100/813) of the provided feedback was not understandable by the model.

**Figure 8.**
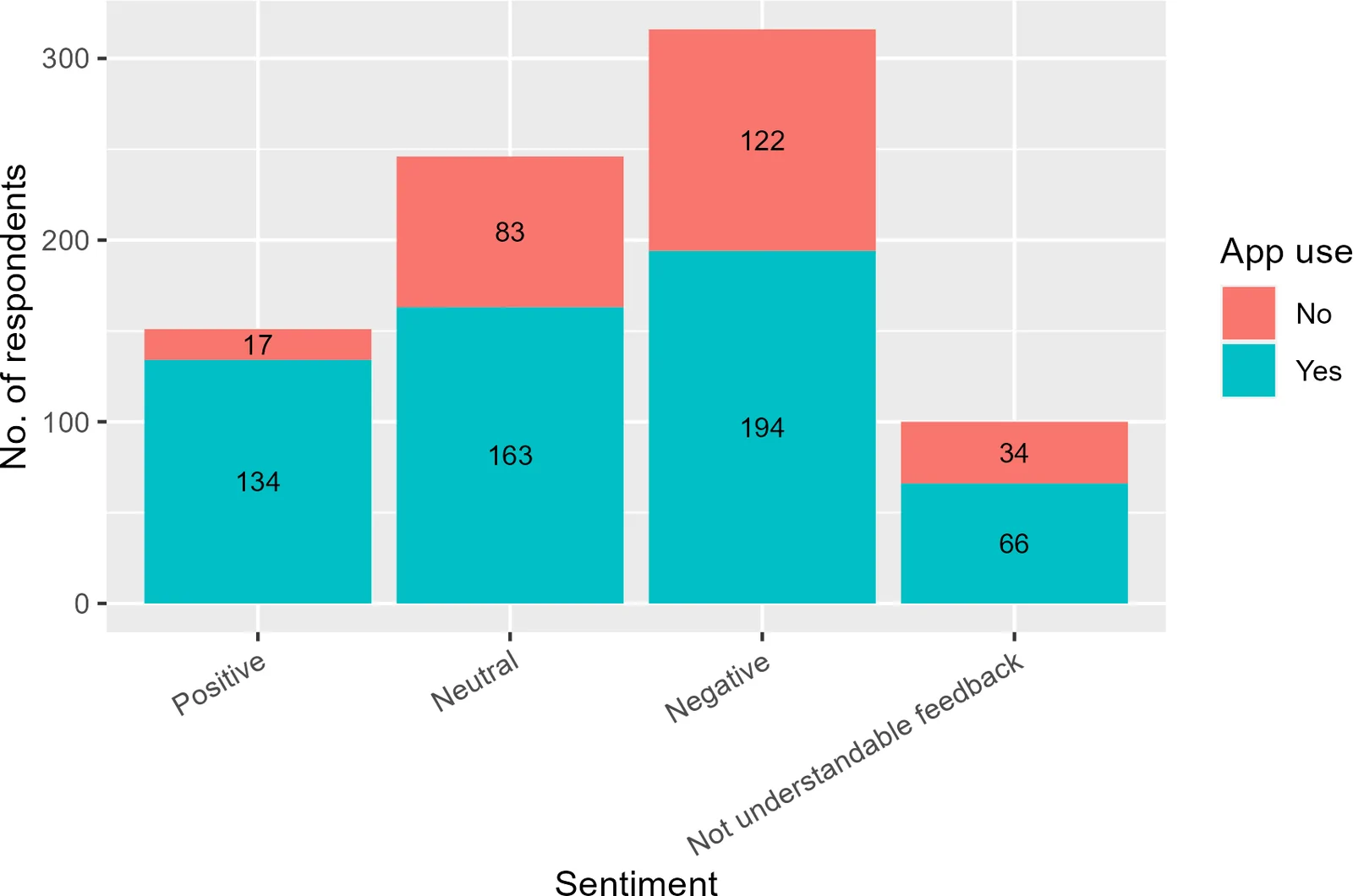
General sentiment analysis of open-ended feedback according to four categories (positive, neutral, negative, and not understandable feedback; n=813).

A higher percentage of positive responses were provided by ENA users than non-ENA users (24%, 134/557 vs 6.6%, 17/256). Nonuser comments more often expressed negative or neutral sentiments (48%, 122/256 and 32%, 83/256, respectively) than ENA user comments (35%, 194/557, and 29%, 163/557, respectively; Figure 8).

## Discussion

### Principal Findings

This study evaluated the Finnish ENA Koronavilkku using predefined KPIs related to uptake, user characteristics, behavioral influence, and acceptability. We found that the ENA achieved high initial uptake but declined over time, with relatively high engagement among users who received tokens. Survey results indicated differences in ENA use across demographic groups and higher self-reported adherence to guidance among ENA users compared to nonusers. While exposure notifications were associated with changes in adherence among some users, barriers such as token issuance and declining engagement over time limited overall system performance.

### Comparison With Prior Work

#### Extent of ENA Use

The Koronavilkku ENA saw widespread adoption during its initial months, with peak active coverage reaching approximately 53% of smartphone users and 45% of the general population. However, as the pandemic progressed, token use declined, especially among people diagnosed with COVID-19, a key target group, with fewer than half of notified COVID-19 cases registered in the ENA, and this proportion fell over time. This trend may reflect reduced app use, reduced token issuance, or a combination of both. Despite this, overall user engagement remained relatively high throughout the ENA’s operational period.

ENA effectiveness is closely tied to its level of adoption. Studies indicate that even modest uptake (15%‐30%) can reduce infections [5,6], while others provide more conservative estimates, indicating that 70%‐80% coverage among smartphone users (56% in the general population) is required to control the pandemic effectively [7,8]. Achieving 80% ENA coverage has been linked to a reduction in COVID-19 mortality by up to 85% [9]. Despite these varying results, one could extrapolate that ENA effectiveness is complex and influenced by multiple factors, with partial benefits seen already at lower coverage rates of around 20% [10]. Notably, Koronavilkku achieved the highest active ENA user coverage among European Union and European Economic Area countries [19].

#### Barriers and Enablers to ENA Use

Our findings identified demographic disparities in ENA use. Males, individuals 35 or older, and those living alone were less likely to use the ENA, while females, those with higher education, and households with children were more likely to be ENA users. However, studies from other European countries revealed differing and sometimes contradictory results. A French study found no significant link between sociodemographic factors and ENA use, except for financial deprivation [12], while a German study reported that males and those 65 years and older were more likely to use the national ENA [13]. Results from the latter study associating higher education levels with increased ENA use were in line with our findings [13]. On the other hand, a different German study indicated that those who were more unwilling to install the national ENA and use it correctly were men and those aged between 30 and 59 years old [14]. These differences suggest that country-specific cultural and contextual differences also play a significant role in ENA use.

A major barrier to the ENA’s performance was token issuance. Over two-thirds of COVID-19-positive users did not receive a token in connection with contact tracing by health care professionals. However, among those who did, most entered their token into the ENA, suggesting strong user motivation when the system functioned properly. Initially, tokens were issued manually by health care contact tracing teams, but high workloads likely caused delays. Recognizing this, Finland implemented partial automation of token issuance in some regions between June and October 2021 [4]. Despite this, issuing exposure tokens remained a significant challenge, particularly during high-incidence periods such as the Omicron wave in late 2021-early 2022. Wider automation of token issuance based on positive results from local systems or a national patient data repository instead of providing a separate system to health care providers would have likely increased the number of issued tokens and shortened token delivery times. However, these possibilities were constrained by a lack of readily available system integrations, risks of not getting complete and reliable result data from the national repository, and short validity periods of tokens. The process for automated token issuance has been described in detail elsewhere [4].

Timely exposure notifications are crucial for effective mitigation. Ferretti et al [20] found that reducing quarantine delays from three to two days significantly curtailed SARS-CoV-2 spread, provided a large portion of the population used the national ENA. Similarly, a Swiss study showed that ENA users quarantined earlier than those not warned by the ENA, though delays in token issuance, linked to manual contact tracing bottlenecks, remained a challenge [16]. A study from Singapore demonstrated that automating public health responses expedited the issuance of quarantine orders compared to manual contact tracing [17].

Our study also explored reasons for nonactivation of the ENA. Consistent with findings from other studies, the most commonly cited reason for not using or uninstalling the ENA was a perceived lack of usefulness [15,21]. Additionally, concerns about battery drain were mentioned as a reason for removing the ENA [21]. These barriers emphasize the need for improved communication around the ENA’s intended benefits and for technical solutions to mitigate the effect on battery life.

#### ENA Influence on User Behavior

Most users who received exposure notifications followed at least some pandemic guidelines recommended by the ENA. ENA users reported adherence at least part-time to general pandemic guidance more often than nonusers, which may reflect self-selection, with ENA users being more health-conscious in general compared to those who did not download the ENA.

Notifications were associated with higher reported adherence among partially compliant users but showed little association among noncompliant individuals. A UK study similarly found high self-reported compliance with self-isolation recommendations following exposure notifications [22].

The only recommendation with declining adherence was “working from home.” This likely reflects job constraints (remote work was not feasible for all occupations) or legal arrangements under which official isolation or quarantine was treated as sick leave, with no obligation to work remotely, even though it was highly recommended by the government [23,24]. Over half of notified users sought health care guidance, which may indicate the ENA played a role in prompting further preventive actions. However, about 20% cited a negative COVID-19 test result as their reason for not fully adhering to guidelines.

#### General Acceptability of the ENA

The ENA’s general acceptability was relatively high, with 68% of respondents using it at least once. However, nearly half eventually stopped using it, particularly as the pandemic prolonged. Similarly, users from other countries also reported their ENAs as trustworthy, reliable, and user-friendly [25]. Nevertheless, a study in Belgium found that features focused on informing citizens or facilitating test appointments were viewed more favorably than those related to access and control [15]. In addition, ENA acceptability has been linked to trust in government, scientists, and health care professionals [12].

A substantial proportion of users expressed interest in enhanced features such as automated exposure notifications to health care providers and improved data tracking. However, it is crucial to balance these functionalities with user privacy concerns that can also evolve over time. In the development of the Finnish ENA, addressing such concerns was a priority. At no point did the ENA process or have access to the identities of users or of persons they encountered, irrespective of infection status. Furthermore, location data from encounters were neither collected nor used, despite the technical feasibility of doing so. Notably, a German study found that public acceptance of privacy-invading technologies, such as tracking, was higher at the beginning of the pandemic, but this acceptance waned as the pandemic continued [26].

Our sentiment analysis of open-ended feedback suggests that users generally expressed more positive attitudes toward the ENA compared to nonusers. While a considerable portion of the feedback reflected neutral or negative sentiments, this should be interpreted with caution. Research on response bias suggests that people are more likely to provide open-ended feedback when they have strong opinions or concerns (either very positive or very negative), whereas users with more moderate views may be less inclined to respond without specific prompting [27-29]. As a result, the overall sentiment of the user base may be more positive than the open-ended responses alone suggest. Additionally, there appear to be differences between those who chose to provide open feedback and the broader survey respondents, suggesting that certain demographics were more inclined to share their views. In contrast, a study conducted in Ireland found that the overall perception of their COVID-19 ENA was mostly positive (90%), although they conducted their sentiment analysis manually [30].

### Limitations

Several limitations should be acknowledged and considered when interpreting the observed results. First, the survey was conducted late in the pandemic, when at-home testing with over-the-counter SARS-CoV-2 diagnostic tests had become more common [31,32]. At that time, laboratory testing was limited [31,32], and few exposure tokens were being issued due to the requirement for a laboratory-confirmed result. Additionally, the ENA was discontinued on June 1, 2022, but many users had already uninstalled it in the preceding months, which may have introduced recall bias. Furthermore, we were only able to analyze prevalence in this cross-sectional study, not infer causal relationships. Although the survey sample represents a small proportion of the Finnish population, weighting was applied based on age, sex, and region to improve representativeness. However, as participation was voluntary, the sample may overrepresent individuals with higher interest in health or digital tools, potentially limiting generalizability. Social desirability bias could have influenced responses to seem more positive, particularly regarding adherence to pandemic guidelines. Despite this, similar patterns of ENA adoption and user behavior have been reported in other European countries, including Germany and the United Kingdom, where uptake and use were associated with sociodemographic factors, trust in public health systems, and user perceptions [13,22].

The LLM used for sentiment analysis has its own drawbacks. First, the model was unable to categorize all responses, with some classified as “not understandable feedback.” Second, each response was assigned a single sentiment category, even though some answers may have contained a mix of sentiments. Third, it faced challenges in accurately interpreting tone and complex language structures. Furthermore, while recent advancements have significantly improved the model’s performance with the Finnish language, translation and interpretation issues can still occur.

The survey did not collect information on the reasons for not using the ENA, only reasons for not activating it after downloading. This additional information could have been useful to identify potential problem areas and opportunities for improvement.

Finally, questions about adherence to health guidelines after receiving an exposure notification referred only to the most recent notification, limiting our ability to assess changes over time. Our analysis relied on a proxy, comparing general behavior during the pandemic to behavior following an exposure notification, but this approach does not provide a comprehensive picture of behavioral shifts.

### Conclusions and Recommendations

This study provides insights into the implementation and performance of a national ENA during a prolonged public health emergency.

Based on observed ENA use trends, regular information campaigns are recommended to maintain long-term public interest and engagement in ENAs during prolonged emergencies.

Furthermore, tailored communication should be developed to encourage uptake among demographic groups with lower adoption rates. Initial studies during ENA rollout could inform these strategies.

Earlier and more systematic automation of token distribution across all regions is critical to enable timely exposure notifications and reduce manual bottlenecks. This should be facilitated by ensuring integrated availability of high-quality laboratory results data from systems using open standard interfaces instead of having to create such mechanisms during the crisis.

Future ENAs should define evaluation criteria during development, with periodic assessments to measure effectiveness and inform improvements. Collecting data on reasons for not downloading the ENA, as well as the timing of installations and removals, could offer valuable insights into retention patterns. Surveys should include questions on factors that would encourage installation, reinstallation, and positive feedback to identify best practices.

From a hardware perspective, nonphone-based contact tracing solutions, such as wearable devices or hardware tokens, could help address battery-life limitations and potentially improve uptake. A deeper analysis of open-ended feedback provided for the Koronavilkku ENA using more advanced language models could provide additional insights into user perceptions and concerns.

Overall, these findings highlight that the success of digital contact tracing tools depends not only on technological functionality, but also on system integration, public trust, and sustained user engagement. Addressing these factors will be essential to support their effective use in future pandemic preparedness and response efforts.

## Supplementary material

10.2196/89472 Multimedia Appendix 1 Key performance indicator metrics.

10.2196/89472 Multimedia Appendix 2 Sentiment analysis validation, exposure notification app use, adherence to behavioral recommendations, and attitudes toward the exposure notification app.

## Abbreviations

ENA: exposure notification app
ERV: exposure risk value
KPI: key performance indicator
LLM: large language model
NIDR: National Infectious Diseases Register
OR: odds ratio
PR: prevalence ratio
