# Supplementary material for: Evaluation of the Finnish Exposure Notification App “Koronavilkku” According to Key Performance Indicators: Cross-Sectional Study
Source: JMIR Public Health Surveill. 2026 Sep 21;12:e89472. doi: 10.2196/89472 (PMC13592390; doi:10.2196/89472)
Supplement: Supplementary file 1 [file publichealth-v12-e89472-s001.pdf]

Key performance indicator: measurement metrics	Definition of metric	Method of calculation	Considerations	Data source (reporting frequency)*	Population	Time period for which data was collected
**a. Extent of ENA use**						
i. Proportion of the population that have downloaded the ENA	Coverage of ENA in the population. The higher the coverage, the more effective the ENA is likely to be in terms of reducing transmission.	1) Number of dowloads/Total population<br>2) Number of dowloads/Total population over 16-years-old	-People may have downloaded the ENA more than once.<br>-Age group over 16-years-old in denominator is used as a proxy for the population that owns a smartphone<br>-The value of this indicator becomes weaker as the pandemic progresses, because the ENAs can be deleted and re-downloaded multiple times, and because a download does not translate into active use.	-Population data (yearly)<br>-Population data over 16 (weekly)<br>-ENA data (weekly)	-Whole of Finnish population (~5.5 mln people)	-Data on number of ENA downloads was collected from week 36-2020 to week 05-2021.
ii. Proportion of the population that actively uses ENA	Coverage of active ENA use in the population. The higher the coverage, the more effective the ENA is likely to be in terms of reducing transmission.	1) Number of active ENA users/Total population<br>2) Number of active ENA users/Total population over 16-years-old	-People may have downloaded the ENA on more than one device.<br>-Age group over 16-years-old in denominator is used as a proxy for the population that owns a smartphone.<br>-Accounting for the fact that some ENAs may be in flight mode and or without access to the Internet for an extended period of time, the numbers are only an approximation and represent a conservative estimation (underestimation).	-Population data (yearly)<br>-Population data over 16 (weekly)<br>-ENA data (weekly)	-Whole of Finnish population (~5.5 mln people)	-Data on number of active ENA users was collected from week 50-2020 to week 16-2022.
iii. Proportion of positive tests that occur among ENA users	Indicates ENA usage/ENA distribution among positive cases.	Number of tokens used/Number of all positive SARS-CoV-2 tests reported by national public health authority	-Tokens generally were not issued automatically in Finland (this was implemented very late and only in certain regions).<br>-The number of tokens entered into the ENA is used as a proxy for the number of positive SARS-CoV-2 tests among ENA users. However this could underestimate the true proportion since some cases might not upload their token.	-COVID-19 data (weekly)<br>-ENA data (weekly)	-Whole of Finnish population (~5.5 mln people)	-Data on number of tokens used/issued was collected from week 37-2020 to week 21-2022.
iv. Proportion of positive tests uploaded into ENA	Indicates proportion of ENA users who enter positive SARS-CoV-2 test result in the ENA. A higher user engagement following a positive test suggests that more people are reached and warned, and further virus transmission is avoided.	Number of tokens used/Number of tokens issued	-Tokens could only be used for 4 hours after being issued (later extended to 12 hours). If people missed the deadline, then a person could ask for a second code, but there is no data on how often this happened.	-ENA data (weekly)	-Whole of Finnish population (~5.5 mln people)	-Data on number of tokens used/issued was collected from week 37-2020 to week 21-2022.
v. Rate of positive tests among ENA users relative to the rate of positive tests reported in the general population	Incidence rate of COVID-19 among ENA users relative to incidence rate in the general population. Indicates if digital proximity tracing notification cascade scaled with rising SARS-CoV-2 incidence.	Number of tokens issued/100 000 active users<br>vs.<br>Number of positive SARS-CoV-2 tests reported by national public health authority/100 000 population	-Tokens could only be used for 4 hours after being issued (later extended to 12 hours). If people missed the deadline, then a person could ask for a second code, but there is no data on how often this happened.	-Population data (yearly)<br>-COVID-19 data (weekly)<br>-ENA data (weekly)	-Whole of Finnish population (~5.5 mln people)	-Data on number of tokens used/issued was collected from week 37-2020 to week 21-2022.
**b. Enablers/barriers to ENA usage**						
i. Sociodemographic characteristics of ENA users vs. non-ENA users	Identification of differences in sociodemographic characteristics between ENA users vs. non-ENA users.	Frequency distribution and prevalence ratios (PR) of sociodemographic characteristics of ENA users vs. non-ENA users. PRs, 95%CIs, and p-values were estimated using log-binomial regression models.	-Sociodemographic characteristics analyzed: sex, age group, region, education, occupation, household situation.	-Survey data	-sample of 4 061 survey respondents	-Data from Koronavilkku cross-sectional survey conducted from April 13 – 22, 2022.
ii. Reasons for non-activation of the ENA	Identification of reasons for not using the ENA	Frequency distribution of reasons associated with non-use of ENA	-	-Survey data	-sample of 4 061 survey respondents	-Data from Koronavilkku cross-sectional survey conducted from April 13 – 22, 2022.
iii. General behaviour during pandemic of ENA users vs. non-ENA users	Identification of differences in general adherence to exposure notification guidance of ENA users vs. non-ENA users during the COVID-19 pandemic	Frequency distribution and prevalence ratios (PR) of adherence to guidance of ENA users vs. non-ENA users. PRs, 95%CIs, and p-values were estimated using log-binomial regression models.	-Social desirability bias might play a role in overestimating the adherence to the guidance.	-Survey data	-sample of 4 061 survey respondents	-Data from Koronavilkku cross-sectional survey conducted from April 13 – 22, 2022.
iv. Token use	Identification of difficulties in using the token	Frequency distribution of token receipt, speed of token receipt, and entering the token into the ENA.	-	-Survey data	-sample of 4 061 survey respondents	-Data from Koronavilkku cross-sectional survey conducted from April 13 – 22, 2022.
**c. Behaviour influence**						

i. Adherence to recommendations after notification	Indicates if and which exposure notification guidance was followed by ENA users after receiving notification.	Frequency distribution of adherence exposure notification guidance by type and level of adherence	-Question in the survey asked only about latest notification.	-Survey data	-sample of 4 061 survey respondents	-Data from Koronavilkku cross-sectional survey conducted from April 13 – 22, 2022.
ii. Change in adherence to recommendations in general during pandemic vs. after notification	Indicates change in behaviour following receipt of an exposure notification.	Differences in adherence levels during the pandemic in general versus after receiving an exposure notification were assessed using Bowker's test of symmetry for paired categorical data. Responses of "unsure" were treated as missing and excluded from the analysis.<br><br>Transition odds were calculated from paired responses by comparing directional changes between adherence categories (e.g., from "part time" to "entire time" vs from "entire time" to "part time"). For each pair of categories, the odds were calculated as the ratio of discordant transitions, restricted to individuals who changed between the two categories. All three adherence categories ("entire time," "part time," "not at all") were retained without collapsing.	-Question in the survey asked only about latest notification.	-Survey data	-sample of 4 061 survey respondents	-Data from Koronavilkku cross-sectional survey conducted from April 13 – 22, 2022.
iii. Reasons for discontinuation of adherence to recommendations	Indicates reasons for discontinuation of adherence to guidance.	Frequency distribution of discontinuation of adherence to guidance.	-Social desirability bias might play a role in underestimating the discontinuation of adherence to the guidance.	-Survey data	-sample of 4 061 survey respondents	-Data from Koronavilkku cross-sectional survey conducted from April 13 – 22, 2022.
iv. Contacting healthcare provider	Indicates healthseeking behaviour facilitated by ENA notification	Frequency distribution of contact with healthcare provider by means of communication.	-	-Survey data	-sample of 4 061 survey respondents	-Data from Koronavilkku cross-sectional survey conducted from April 13 – 22, 2022.
**d. Acceptability**						
i. ENA use	Indicates percentage of ENA users, non-ENA users, as well as user retention.	Frequency distribution of dowloading, activating, using, and removing the ENA.	-	-Survey data	-sample of 4 061 survey respondents	-Data from Koronavilkku cross-sectional survey conducted from April 13 – 22, 2022.
ii. Reasons for removal	Identification of reasons for removing the ENA	Frequency distribution of reasons associated with removal of ENA	-	-Survey data	-sample of 4 061 survey respondents	-Data from Koronavilkku cross-sectional survey conducted from April 13 – 22, 2022.
iii. Overall feedback on ENA	Attitude towards general statements about the ENA	Frequency distribution of statements associated with feedback on ENA among all respondents vs. among ENA users.	-	-Survey data	-sample of 4 061 survey respondents	-Data from Koronavilkku cross-sectional survey conducted from April 13 – 22, 2022.
iv. Sentiment analysis	General sentiment analysis of open question on notification feedback.	Sentiment analysis of open question on notification feedback.	-LLM llama version 3.1 70B was used for the sentiment analysis.<br>-Open feedback was analyzed and divided into four categories: positive, neutral, negative, and not understandable feedback.	-Survey data	-sample of 4 061 survey respondents	-Data from Koronavilkku cross-sectional survey conducted from April 13 – 22, 2022.

*More information about data sources can be found in the "Data collection" section
