# Supplementary material for: Evaluation of the Finnish Exposure Notification App “Koronavilkku” According to Key Performance Indicators: Cross-Sectional Study
Source: JMIR Public Health Surveill. 2026 Sep 21;12:e89472. doi: 10.2196/89472 (PMC13592390; doi:10.2196/89472)
Supplement: Supplementary file 2 [file publichealth-v12-e89472-s002.docx]

# Sentiment analysis validation, ENA use, adherence to behavioral recommendations, and attitudes toward the ENA.

## Table S1. Confusion matrix for sentiment analysis of manual human annotations vs. Llama (random sampling) (n=100).

|  | **Llama: positive** | **Llama: neutral** | **Llama: negative** | **Llama: not understandable** |
| --- | --- | --- | --- | --- |
| Manual: positive (n=26) | 17 | 6 | 1 | 2 |
| Manual: neutral (n=38) | 2 | 18 | 7 | 11 |
| Manual: negative (n=33) | 1 | 4 | 26 | 2 |
| Manual: not understandable (n=3) | 0 | 1 | 0 | 2 |

## Table S2. Weighted frequency distribution of reasons for not activating the ENA (n = 149, weighted N = 163). Data from survey conducted in Finland from April 13 – 22, 2022. Results weighted according to age, sex, and regional distribution of the Finnish population.

| Variable | N | % | N (weighted) | % (weighted) | 95% CI | deff |
| --- | --- | --- | --- | --- | --- | --- |
| I do not have a suitable phone at my disposal | 1 | 0.7% | 1.057117 | 0.6% | 0.1-4.5 | 10.81683 |
| I have concerns about the security of the application | 11 | 7.4% | 11.619268 | 7.1% | 3.9-12.5 | 11.10015 |
| I heard that the app drains the phone battery | 35 | 23% | 39.959973 | 24.4% | 18.0-32.3 | 11.91974 |
| The app would cause me extra worry or stress about my health | 13 | 8.7% | 15.045204 | 9.2% | 5.4-15.3 | 12.02165 |
| I felt safe from infection because where I live there are few infections | 17 | 11% | 19.807240 | 12.1% | 7.6-18.8 | 12.12426 |
| I felt safe from infection because I have been vaccinated or already had COVID-19 | 25 | 17% | 27.303511 | 16.7% | 11.4-23.7 | 11.45728 |
| I felt safe from infection because so many others have been vaccinated | 5 | 3.4% | 5.966857 | 3.6% | 1.5-8.6 | 12.48334 |
| I didn’t move much outside the home | 34 | 23% | 38.600002 | 23.6% | 17.2-31.4 | 11.99509 |
| I don’t think the app is useful to me | 52 | 35% | 55.592020 | 34.0% | 26.7-42.1 | 11.47351 |
| I don’t think the app was significant in epidemic management | 53 | 36% | 57.665391 | 35.3% | 27.9-43.5 | 11.60842 |
| Not interested in the coronavirus epidemic | 11 | 7.4% | 11.422763 | 7.0% | 3.9-12.4 | 11.00511 |
| I don’t know | 8 | 5.4% | 8.735514 | 5.3% | 2.6-10.5 | 11.67398 |
| Other | 11 | 7.4% | 11.849677 | 7.2% | 4.0-12.8 | 11.35018 |

## Table S3. Weighted frequency distribution of adherence to pandemic behavioral recommendations after receiving an exposure notification in the ENA (n = 644, weighted N = 670). Data from survey conducted in Finland from April 13 – 22, 2022. Results weighted according to age, sex, and regional distribution of the Finnish population.

| Variable | label | N | % | N (weighted) | % (weighted) | 95% CI | deff |
| --- | --- | --- | --- | --- | --- | --- | --- |
| Working from home | Entire time | 311 | 48% | 321.62980 | 48.0% | 44.2-51.9 | 26.65225 |
| Working from home | Not at all | 161 | 25% | 166.77831 | 24.9% | 21.7-28.4 | 26.69326 |
| Working from home | Part of the time | 144 | 22% | 151.51541 | 22.6% | 19.5-26.1 | 26.81524 |
| Working from home | Unsure | 28 | 4.3% | 29.61162 | 4.4% | 3.1-6.4 | 27.37294 |
| Self-observation of well-being | Entire time | 386 | 60% | 398.26752 | 59.5% | 55.6-63.3 | 26.76657 |
| Self-observation of well-being | Not at all | 36 | 5.6% | 40.65317 | 6.1% | 4.4-8.3 | 28.71525 |
| Self-observation of well-being | Part of the time | 212 | 33% | 219.95754 | 32.9% | 29.3-36.6 | 26.64273 |
| Self-observation of well-being | Unsure | 10 | 1.6% | 10.65692 | 1.6% | 0.9-2.9 | 27.25190 |
| Avoiding social interactions | Entire time | 309 | 48% | 318.63998 | 47.6% | 43.7-51.5 | 26.65099 |
| Avoiding social interactions | Not at all | 102 | 16% | 108.33521 | 16.2% | 13.5-19.3 | 27.08849 |
| Avoiding social interactions | Part of the time | 216 | 34% | 224.38220 | 33.5% | 29.9-37.3 | 26.61970 |
| Avoiding social interactions | Unsure | 17 | 2.6% | 18.17776 | 2.7% | 1.7-4.4 | 27.63504 |
| Wearing a mask | Entire time | 461 | 72% | 476.92204 | 71.2% | 67.6-74.6 | 26.87232 |
| Wearing a mask | Not at all | 42 | 6.5% | 45.50788 | 6.8% | 5.0-9.1 | 27.76129 |
| Wearing a mask | Part of the time | 127 | 20% | 132.23665 | 19.8% | 16.8-23.0 | 26.70385 |
| Wearing a mask | Unsure | 14 | 2.2% | 14.86858 | 2.2% | 1.3-3.7 | 27.07497 |
| Hand hygiene | Entire time | 442 | 69% | 455.97534 | 68.1% | 64.3-71.6 | 26.86733 |
| Hand hygiene | Not at all | 49 | 7.6% | 51.15154 | 7.6% | 5.8-10.0 | 26.75973 |
| Hand hygiene | Part of the time | 131 | 20% | 138.49104 | 20.7% | 17.7-24.0 | 26.89732 |
| Hand hygiene | Unsure | 22 | 3.4% | 23.91723 | 3.6% | 2.4-5.4 | 27.96430 |
| Avoiding errands | Entire time | 236 | 37% | 245.31530 | 36.6% | 33.0-40.5 | 26.61884 |
| Avoiding errands | Not at all | 218 | 34% | 224.60167 | 33.5% | 30.0-37.3 | 26.58522 |
| Avoiding errands | Part of the time | 165 | 26% | 174.13400 | 26.0% | 22.7-29.6 | 26.95082 |
| Avoiding errands | Unsure | 25 | 3.9% | 25.48418 | 3.8% | 2.6-5.6 | 26.22131 |
| Cough hygiene | Entire time | 468 | 73% | 482.80416 | 72.1% | 68.5-75.5 | 26.97209 |
| Cough hygiene | Not at all | 38 | 5.9% | 41.46903 | 6.2% | 4.5-8.4 | 27.90625 |
| Cough hygiene | Part of the time | 124 | 19% | 130.70695 | 19.5% | 16.6-22.8 | 26.89480 |
| Cough hygiene | Unsure | 14 | 2.2% | 14.55501 | 2.2% | 1.3-3.7 | 26.57326 |

## Table S4. Weighted frequency distribution of reasons for discontinuation or nonadherence to pandemic behavioral recommendations after receiving an exposure notification in the ENA (n = 534, weighted N = 556). Data from survey conducted in Finland from April 13 – 22, 2022. Results weighted according to age, sex, and regional distribution of the Finnish population.

| Variable | N | % | N (weighted) | % (weighted) | 95% CI | deff |
| --- | --- | --- | --- | --- | --- | --- |
| I was placed in quarantine and given further instructions | 105 | 20% | 108.590123 | 19.5% | 16.4-23.2 | 25.91542 |
| The health service contacted me and gave me instructions | 114 | 21% | 118.729553 | 21.4% | 18.1-25.1 | 26.03029 |
| The employer gave differing instructions | 110 | 21% | 113.543926 | 20.4% | 17.2-24.1 | 25.84937 |
| School or college gave differing instructions | 57 | 11% | 59.576208 | 10.7% | 8.3-13.7 | 26.11337 |
| The advice phoneline gave differing instructions | 49 | 9.2% | 50.584558 | 9.1% | 6.9-11.9 | 25.92502 |
| A friend or acquaintance told me that I don’t have to follow the instructions | 30 | 5.6% | 31.900472 | 5.7% | 4.0-8.1 | 26.42311 |
| I got a negative test result | 123 | 23% | 126.735825 | 22.8% | 19.4-26.6 | 26.05511 |
| I did not follow the instructions because I had received a full course of vaccines | 77 | 14% | 81.986891 | 14.8% | 11.9-18.1 | 26.59183 |
| I did not follow the instructions because I had previously had COVID-19 | 33 | 6.2% | 35.154187 | 6.3% | 4.5-8.8 | 26.72182 |
| I did not follow the instructions because I was asymptomatic | 109 | 20% | 115.664370 | 20.8% | 17.5-24.5 | 26.57903 |
| I thought I knew why I received the exposure notification and did not consider it as an actual exposure | 85 | 16% | 88.632999 | 16.0% | 13.1-19.3 | 26.11986 |
| I forgot about it | 23 | 4.3% | 23.497383 | 4.2% | 2.8-6.3 | 25.68205 |
| The issue no longer seemed relevant | 49 | 9.2% | 52.059073 | 9.4% | 7.1-12.2 | 26.62437 |
| I am very critical of vaccination or contact tracing | 22 | 4.1% | 23.614148 | 4.3% | 2.8-6.4 | 26.66392 |
| I have not changed my behaviour during the epidemic | 36 | 6.7% | 39.180412 | 7.1% | 5.1-9.6 | 27.20734 |
| Using common sense works well | 55 | 10% | 58.392578 | 10.5% | 8.1-13.5 | 26.61185 |
| Following the guidelines would have made my everyday life too difficult | 24 | 4.5% | 25.103317 | 4.5% | 3.0-6.7 | 26.26723 |
| I did not understand the instructions | 7 | 1.3% | 7.497376 | 1.3% | 0.6-2.8 | 26.77973 |
| Other | 27 | 5.1% | 27.592911 | 5.0% | 3.4-7.2 | 25.63895 |

## Table S5. Weighted frequency distribution of reasons for discontinuation of ENA use (n = 1370, weighted N = 1444). Data from Koronavilkku survey conducted in Finland from April 13 – 22, 2022. Results weighted according to age, sex, and regional distribution of the Finnish population.

| Variable | N | % | N (weighted) | % (weighted) | 95% CI | deff |
| --- | --- | --- | --- | --- | --- | --- |
| I forgot to reinstall | 106 | 7.7% | 109.27014 | 7.6% | 6.3-9.1 | 19.51500 |
| Concerns about the security of the application | 56 | 4.1% | 58.19819 | 4.0% | 3.1-5.2 | 19.55067 |
| The app was draining my phone’s battery | 356 | 26% | 378.29162 | 26.2% | 23.9-28.6 | 20.28867 |
| The app caused me extra worry or stress about my health | 90 | 6.6% | 91.24404 | 6.3% | 5.2-7.7 | 19.14485 |
| I felt safe from infection because I live in a place where infections are rare | 89 | 6.5% | 93.41320 | 6.5% | 5.3-7.9 | 19.99112 |
| I felt safe from infection because I have been vaccinated or already had the disease | 318 | 23% | 333.46361 | 23.1% | 20.9-25.4 | 20.05694 |
| I felt safe from infection because so many others have been vaccinated | 72 | 5.3% | 74.13745 | 5.1% | 4.1-6.4 | 19.49717 |
| I received exposure notifications, although I believe I should not have received them | 49 | 3.6% | 49.65225 | 3.4% | 2.6-4.5 | 19.01520 |
| I did not receive exposure notifications, although I believe I should have received them | 327 | 24% | 339.45810 | 23.5% | 21.3-25.9 | 19.92873 |
| I did not move much outside the home | 221 | 16% | 238.92037 | 16.5% | 14.6-18.7 | 20.56215 |
| I don’t think the app is useful to me | 444 | 32% | 466.07418 | 32.3% | 29.8-34.8 | 20.09410 |
| I don’t think the app was significant in epidemic management | 472 | 34% | 497.36568 | 34.4% | 31.9-37.0 | 20.13111 |
| Not interested in the coronavirus epidemic | 29 | 2.1% | 29.62992 | 2.1% | 1.4-3.0 | 19.45187 |
| I don’t know | 11 | 0.8% | 11.65342 | 0.8% | 0.4-1.5 | 19.81447 |
| Other | 112 | 8.2% | 113.99870 | 7.9% | 6.6-9.4 | 19.47568 |

## Table S6. Weighted frequency distribution of attitude towards general statements about the ENA among a) all respondents (N = 4061, weighted N = 4334); b) among ENA users (n = 2740, weighted N = 2927). Data from Koronavilkku survey conducted in Finland from April 13 – 22, 2022. Results weighted according to age, sex, and regional distribution of the Finnish population.

## A. All respondents

| Variable | label | N | % | N (weighted) | % (weighted) | 95% CI | deff |
| --- | --- | --- | --- | --- | --- | --- | --- |
| Koronavilkku was a good app | Agree | 816 | 20% | 895.9300 | 20.7% | 19.5-21.9 | 15.77147 |
| Koronavilkku was a good app | Partly agree | 1,159 | 29% | 1237.6036 | 28.6% | 27.2-30.0 | 15.74893 |
| Koronavilkku was a good app | Partly disagree | 664 | 16% | 695.6186 | 16.1% | 14.9-17.2 | 16.11210 |
| Koronavilkku was a good app | Disagree | 660 | 16% | 690.3144 | 15.9% | 14.9-17.1 | 15.39232 |
| Koronavilkku was a good app | Unsure | 762 | 19% | 814.5334 | 18.8% | 17.7-20.0 | 14.47603 |
| If necessary, I would use a similar application in the future | Agree | 1,346 | 33% | 1447.7621 | 33.4% | 32.0-34.8 | 14.24047 |
| If necessary, I would use a similar application in the future | Partly agree | 1,134 | 28% | 1221.7398 | 28.2% | 26.8-29.6 | 16.29285 |
| If necessary, I would use a similar application in the future | Partly disagree | 462 | 11% | 479.7124 | 11.1% | 10.1-12.1 | 15.91701 |
| If necessary, I would use a similar application in the future | Disagree | 596 | 15% | 622.2868 | 14.4% | 13.4-15.4 | 14.27384 |
| If necessary, I would use a similar application in the future | Unsure | 523 | 13% | 562.4989 | 13.0% | 12.0-14.0 | 15.28734 |
| I would like to use a separate small keyfob as the ENA | Agree | 547 | 13% | 594.3386 | 13.7% | 12.7-14.8 | 16.58396 |
| I would like to use a separate small keyfob as the ENA | Partly agree | 709 | 17% | 758.5199 | 17.5% | 16.3-18.7 | 16.44721 |
| I would like to use a separate small keyfob as the ENA | Partly disagree | 663 | 16% | 700.1937 | 16.2% | 15.0-17.3 | 16.22884 |
| I would like to use a separate small keyfob as the ENA | Disagree | 1,457 | 36% | 1524.8877 | 35.2% | 33.7-36.7 | 16.26921 |
| I would like to use a separate small keyfob as the ENA | Unsure | 685 | 17% | 756.0600 | 17.4% | 16.3-18.7 | 16.79976 |
| Information about my own exposure should have been passed on for statistics purposes | Agree | 2,196 | 54% | 2348.5342 | 54.2% | 52.7-55.7 | 15.51952 |
| Information about my own exposure should have been passed on for statistics purposes | Partly agree | 958 | 24% | 1025.1611 | 23.7% | 22.3-25.0 | 16.44371 |
| Information about my own exposure should have been passed on for statistics purposes | Partly disagree | 247 | 6.1% | 259.6881 | 6.0% | 5.3-6.8 | 16.18473 |
| Information about my own exposure should have been passed on for statistics purposes | Disagree | 294 | 7.2% | 305.4061 | 7.0% | 6.3-7.9 | 15.37672 |
| Information about my own exposure should have been passed on for statistics purposes | Unsure | 366 | 9.0% | 395.2105 | 9.1% | 8.3-10.1 | 16.34946 |
| Information about my own exposure should have been passed on to health care | Agree | 2,193 | 54% | 2357.1834 | 54.4% | 52.9-55.9 | 15.62897 |
| Information about my own exposure should have been passed on to health care | Partly agree | 960 | 24% | 1020.7499 | 23.6% | 22.3-24.9 | 16.39313 |
| Information about my own exposure should have been passed on to health care | Partly disagree | 270 | 6.6% | 285.8229 | 6.6% | 5.9-7.4 | 16.14069 |
| Information about my own exposure should have been passed on to health care | Disagree | 269 | 6.6% | 276.3849 | 6.4% | 5.7-7.2 | 15.35824 |
| Information about my own exposure should have been passed on to health care | Unsure | 369 | 9.1% | 393.8589 | 9.1% | 8.2-10.0 | 15.97666 |
| My location data should be used if security can be guaranteed | Agree | 1,689 | 42% | 1813.4145 | 41.8% | 40.4-43.3 | 15.16377 |
| My location data should be used if security can be guaranteed | Partly agree | 1,159 | 29% | 1247.9052 | 28.8% | 27.4-30.2 | 16.53433 |
| My location data should be used if security can be guaranteed | Partly disagree | 396 | 9.8% | 418.7526 | 9.7% | 8.8-10.6 | 16.16394 |
| My location data should be used if security can be guaranteed | Disagree | 511 | 13% | 530.8550 | 12.2% | 11.3-13.3 | 14.86091 |
| My location data should be used if security can be guaranteed | Unsure | 306 | 7.5% | 323.0727 | 7.5% | 6.7-8.3 | 15.92631 |
| I would have liked to receive more detailed information about the exposures | Agree | 1,343 | 33% | 1413.4051 | 32.6% | 31.2-34.0 | 15.39592 |
| I would have liked to receive more detailed information about the exposures | Partly agree | 1,184 | 29% | 1272.7168 | 29.4% | 28.0-30.8 | 16.50162 |
| I would have liked to receive more detailed information about the exposures | Partly disagree | 364 | 9.0% | 386.1833 | 8.9% | 8.1-9.8 | 16.22392 |
| I would have liked to receive more detailed information about the exposures | Disagree | 407 | 10% | 424.3913 | 9.8% | 8.9-10.7 | 15.26503 |
| I would have liked to receive more detailed information about the exposures | Unsure | 763 | 19% | 837.3035 | 19.3% | 18.1-20.6 | 16.59041 |
| If given the option, I would have generated a token myself based on a positive home test | Agree | 1,335 | 33% | 1401.0640 | 32.3% | 30.9-33.8 | 15.18201 |
| If given the option, I would have generated a token myself based on a positive home test | Partly agree | 1,076 | 26% | 1160.9168 | 26.8% | 25.4-28.2 | 16.49223 |
| If given the option, I would have generated a token myself based on a positive home test | Partly disagree | 316 | 7.8% | 339.1371 | 7.8% | 7.0-8.7 | 16.44073 |
| If given the option, I would have generated a token myself based on a positive home test | Disagree | 409 | 10% | 429.5129 | 9.9% | 9.0-10.9 | 15.52674 |
| If given the option, I would have generated a token myself based on a positive home test | Unsure | 925 | 23% | 1003.3691 | 23.2% | 21.9-24.5 | 16.13345 |

## B. ENA users

| Variable | label | N | % | N (weighted) | % (weighted) | 95% CI | deff |
| --- | --- | --- | --- | --- | --- | --- | --- |
| Koronavilkku was a good app | Agree | 743 | 27% | 812.6379 | 27.8% | 26.1-29.5 | 16.43779 |
| Koronavilkku was a good app | Partly agree | 971 | 35% | 1030.2337 | 35.2% | 33.4-37.0 | 16.17125 |
| Koronavilkku was a good app | Partly disagree | 488 | 18% | 509.5756 | 17.4% | 16.0-18.9 | 15.86770 |
| Koronavilkku was a good app | Disagree | 292 | 11% | 307.3776 | 10.5% | 9.4-11.7 | 15.91769 |
| Koronavilkku was a good app | Unsure | 246 | 9.0% | 267.1980 | 9.1% | 8.1-10.3 | 16.52858 |
| If necessary, I would use a similar application in the future | Agree | 1,246 | 45% | 1338.7260 | 45.7% | 43.8-47.6 | 16.22031 |
| If necessary, I would use a similar application in the future | Partly agree | 880 | 32% | 943.3204 | 32.2% | 30.5-34.0 | 16.25641 |
| If necessary, I would use a similar application in the future | Partly disagree | 273 | 10.0% | 282.0844 | 9.6% | 8.6-10.8 | 15.65842 |
| If necessary, I would use a similar application in the future | Disagree | 167 | 6.1% | 177.8563 | 6.1% | 5.2-7.0 | 16.07021 |
| If necessary, I would use a similar application in the future | Unsure | 174 | 6.4% | 185.0356 | 6.3% | 5.5-7.3 | 16.18851 |
| I would like to use a separate small keyfob as the ENA | Agree | 401 | 15% | 438.4376 | 15.0% | 13.7-16.4 | 16.46489 |
| I would like to use a separate small keyfob as the ENA | Partly agree | 480 | 18% | 514.3458 | 17.6% | 16.2-19.1 | 16.19334 |
| I would like to use a separate small keyfob as the ENA | Partly disagree | 503 | 18% | 530.7451 | 18.1% | 16.7-19.6 | 16.07413 |
| I would like to use a separate small keyfob as the ENA | Disagree | 971 | 35% | 1017.0370 | 34.7% | 33.0-36.6 | 16.03624 |
| I would like to use a separate small keyfob as the ENA | Unsure | 385 | 14% | 426.4572 | 14.6% | 13.3-16.0 | 16.85560 |
| Information about my own exposure should have been passed on for statistics purposes | Agree | 1,711 | 62% | 1829.2614 | 62.5% | 60.6-64.3 | 16.20752 |
| Information about my own exposure should have been passed on for statistics purposes | Partly agree | 609 | 22% | 649.1714 | 22.2% | 20.6-23.8 | 16.20055 |
| Information about my own exposure should have been passed on for statistics purposes | Partly disagree | 155 | 5.7% | 161.8067 | 5.5% | 4.7-6.5 | 15.78246 |
| Information about my own exposure should have been passed on for statistics purposes | Disagree | 101 | 3.7% | 107.6001 | 3.7% | 3.0-4.5 | 15.99906 |
| Information about my own exposure should have been passed on for statistics purposes | Unsure | 164 | 6.0% | 179.1831 | 6.1% | 5.3-7.1 | 16.74735 |
| Information about my own exposure should have been passed on to health care | Agree | 1,693 | 62% | 1816.8955 | 62.1% | 60.2-63.9 | 16.15291 |
| Information about my own exposure should have been passed on to health care | Partly agree | 620 | 23% | 657.3328 | 22.5% | 20.9-24.1 | 16.12601 |
| Information about my own exposure should have been passed on to health care | Partly disagree | 171 | 6.2% | 182.1169 | 6.2% | 5.4-7.2 | 15.97485 |
| Information about my own exposure should have been passed on to health care | Disagree | 99 | 3.6% | 103.9597 | 3.6% | 2.9-4.3 | 15.93034 |
| Information about my own exposure should have been passed on to health care | Unsure | 157 | 5.7% | 166.7178 | 5.7% | 4.9-6.6 | 16.18345 |
| My location data should be used if security can be guaranteed | Agree | 1,408 | 51% | 1512.3587 | 51.7% | 49.8-53.6 | 16.20086 |
| My location data should be used if security can be guaranteed | Partly agree | 803 | 29% | 860.2294 | 29.4% | 27.7-31.2 | 16.24559 |
| My location data should be used if security can be guaranteed | Partly disagree | 227 | 8.3% | 237.9458 | 8.1% | 7.2-9.2 | 15.87255 |
| My location data should be used if security can be guaranteed | Disagree | 174 | 6.4% | 182.5196 | 6.2% | 5.4-7.2 | 15.79345 |
| My location data should be used if security can be guaranteed | Unsure | 128 | 4.7% | 133.9691 | 4.6% | 3.8-5.4 | 16.05062 |
| I would have liked to receive more detailed information about the exposures | Agree | 1,122 | 41% | 1178.2236 | 40.3% | 38.4-42.1 | 16.08131 |
| I would have liked to receive more detailed information about the exposures | Partly agree | 845 | 31% | 905.1613 | 30.9% | 29.2-32.7 | 16.25611 |
| I would have liked to receive more detailed information about the exposures | Partly disagree | 223 | 8.1% | 238.8947 | 8.2% | 7.2-9.3 | 16.17281 |
| I would have liked to receive more detailed information about the exposures | Disagree | 143 | 5.2% | 151.5937 | 5.2% | 4.4-6.1 | 16.02781 |
| I would have liked to receive more detailed information about the exposures | Unsure | 407 | 15% | 453.1494 | 15.5% | 14.1-16.9 | 16.94735 |
| If given the option, I would have generated a token myself based on a positive home test | Agree | 1,137 | 41% | 1193.6506 | 40.8% | 38.9-42.7 | 16.09766 |
| If given the option, I would have generated a token myself based on a positive home test | Partly agree | 785 | 29% | 847.0412 | 28.9% | 27.2-30.7 | 16.32297 |
| If given the option, I would have generated a token myself based on a positive home test | Partly disagree | 192 | 7.0% | 203.3362 | 6.9% | 6.0-8.0 | 16.00696 |
| If given the option, I would have generated a token myself based on a positive home test | Disagree | 154 | 5.6% | 164.9195 | 5.6% | 4.8-6.6 | 16.25921 |
| If given the option, I would have generated a token myself based on a positive home test | Unsure | 472 | 17% | 518.0753 | 17.7% | 16.3-19.2 | 16.64315 |
